# Supplementary material for: Pan-Genome-Based Analysis as a Framework for Demarcating Two Closely Related Methanotroph Genera Methylocystis and Methylosinus
Source: Microorganisms. 2020 May 20;8(5):768. doi: 10.3390/microorganisms8050768 (PMC7285482; doi:10.3390/microorganisms8050768)
Supplement: Supplementary file 1 [file microorganisms-08-00768-s001.zip › Suppl_material/Pan-genome_supplementary material.docx]

**Pan-genome-based analysis as a framework for demarcating two closely related methanotroph genera *Methylocystis* and *Methylosinus***

Igor Y. Oshkin^1^*, Kirill K. Miroshnikov^1^, Denis S. Gruzdev^2^, Svetlana N. Dedysh^1^

^1^*Winogradsky Institute of Microbiology, Research Center of Biotechnology of the Russian Academy of Sciences, Moscow, 119071, Russia*

^2^*Institute of Bioengineering, Research Center of Biotechnology of the Russian Academy of Sciences, Moscow, 119071, Russia*

*Author for Correspondence: Igor Y. Oshkin, Winogradsky Institute of Microbiology, Research Center of Biotechnology of the Russian Academy of Sciences, +7-(499)-135-0591, +7-(499)-135-6530, ig.owkin@gmail.com

**Running title:** Pan-genome analysis of the *Methylocystis*/*Methylosinus* group


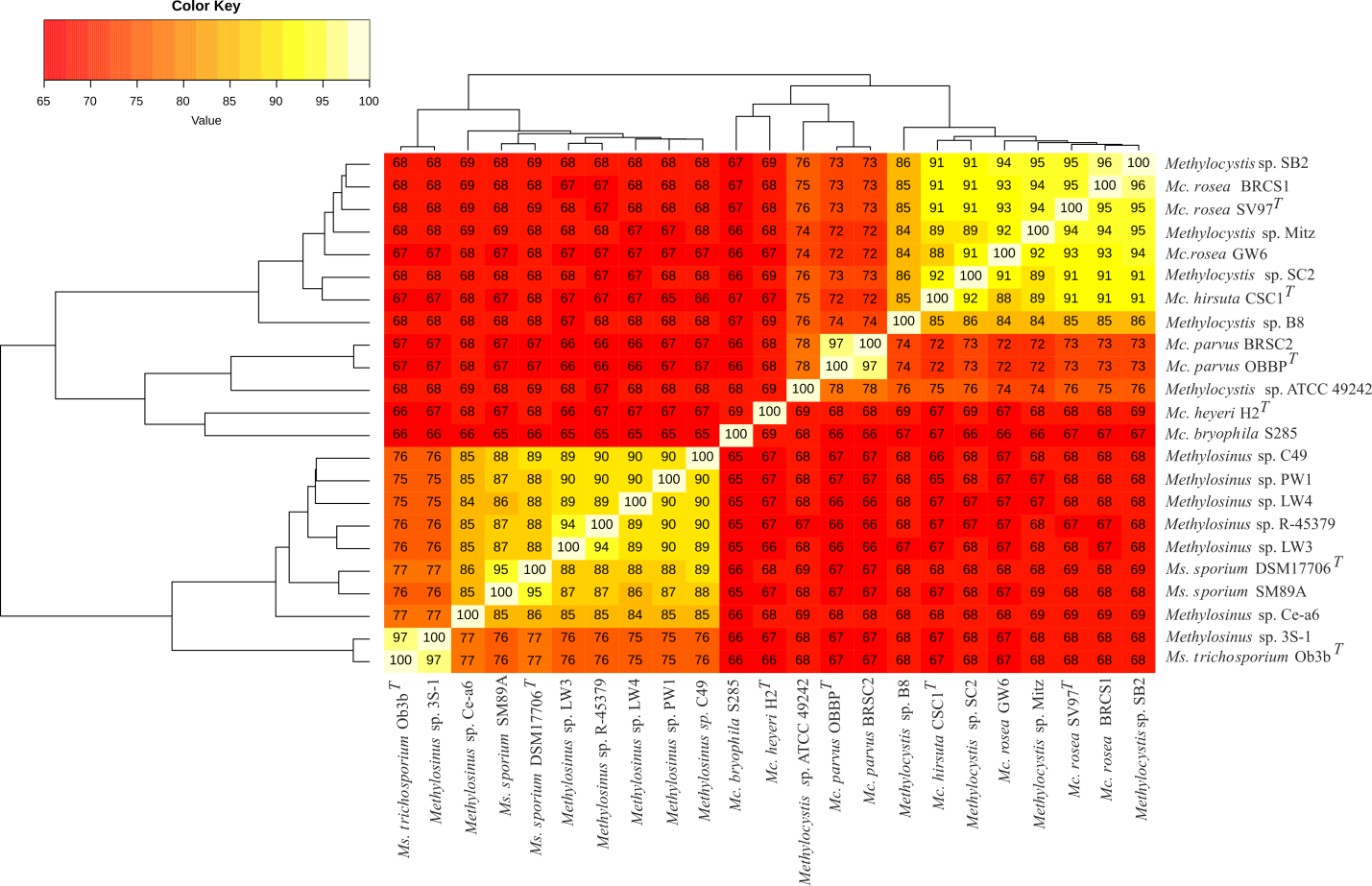


Figure S1. Heatmap derived from an Average Nucleotide Identity (ANI) matrix calculated for each pair of the genomes of *Methylocystis/Methylosinus* group.


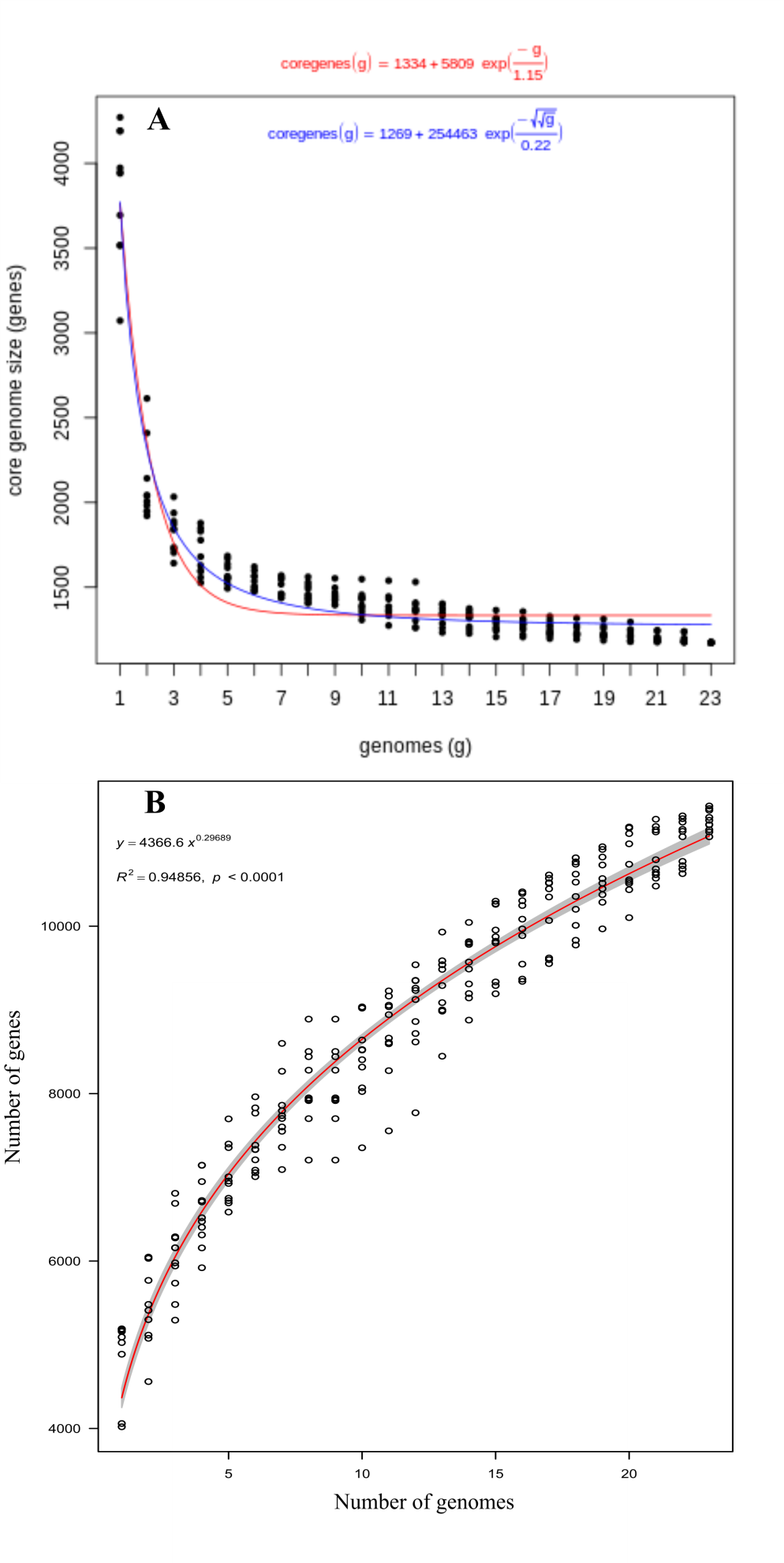


Figure S2. The *Methylocystis/Methylosinus* core genome (A) and pan-genome (B) as a function of the number of genomes included.


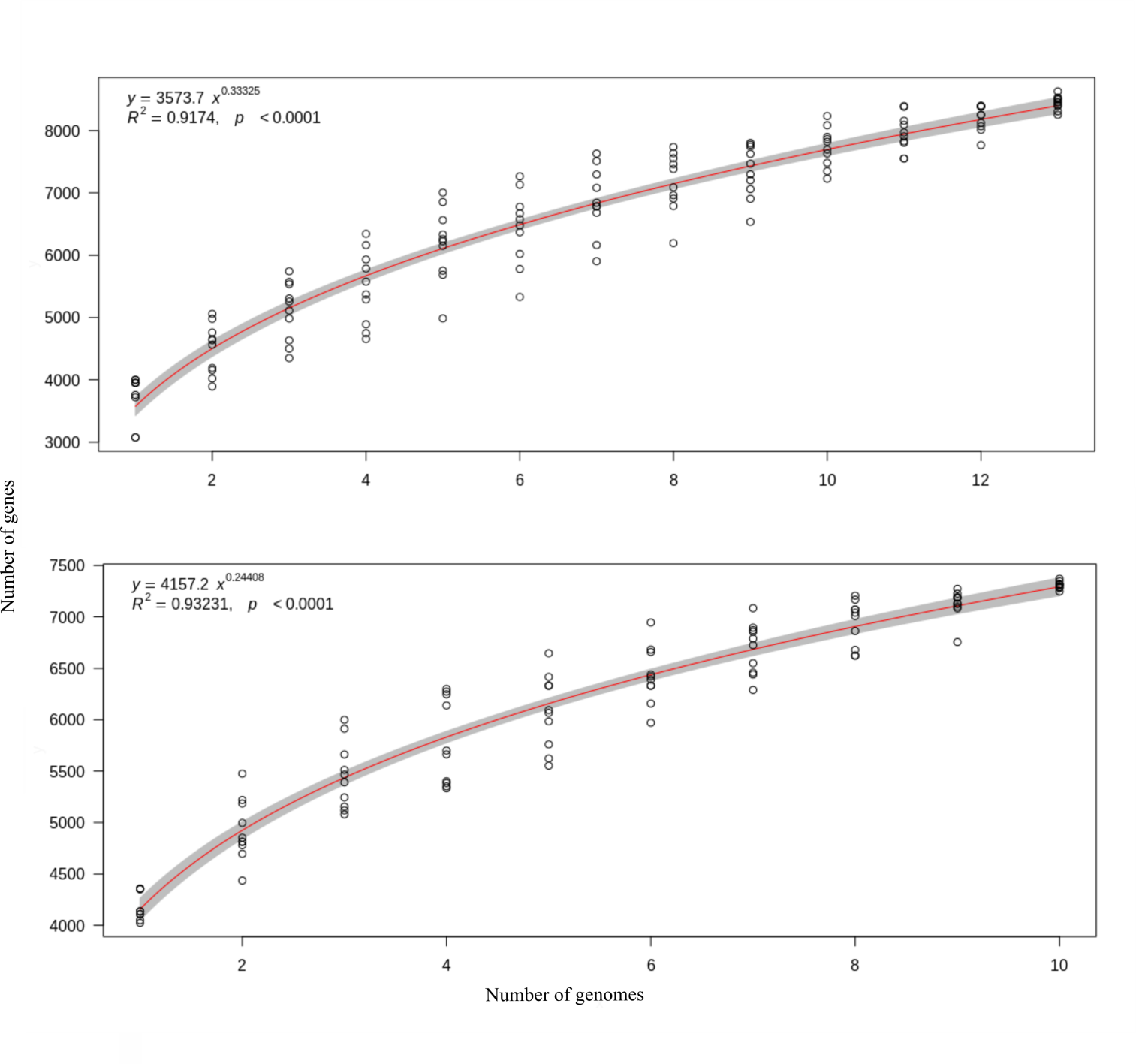


Figure S3. The *Methylocystis* pan-genome (A) and *Methylosinus* pan-genome (B) as a function of the number of genomes included (V1–V24).


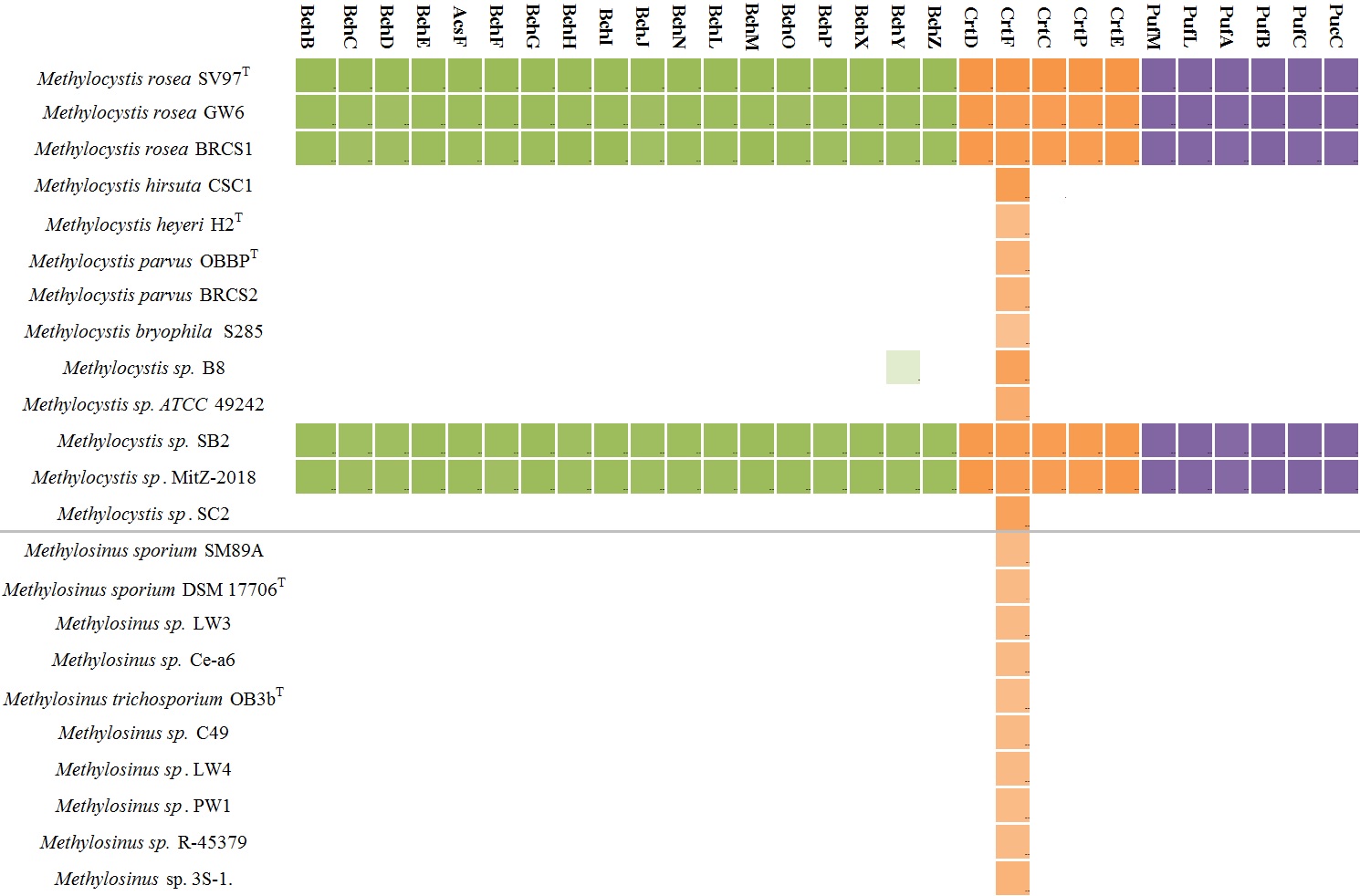


Figure S4. Heatmap displaying distribution of phototrophy-related genes (green) and carotenoids biosynthesis genes (orange).
