# Supplementary figures and images for: Pan-Genome-Based Analysis as a Framework for Demarcating Two Closely Related Methanotroph Genera Methylocystis and Methylosinus
Source: Microorganisms. 2020 May 20;8(5):768. doi: 10.3390/microorganisms8050768 (PMC7285482; doi:10.3390/microorganisms8050768)

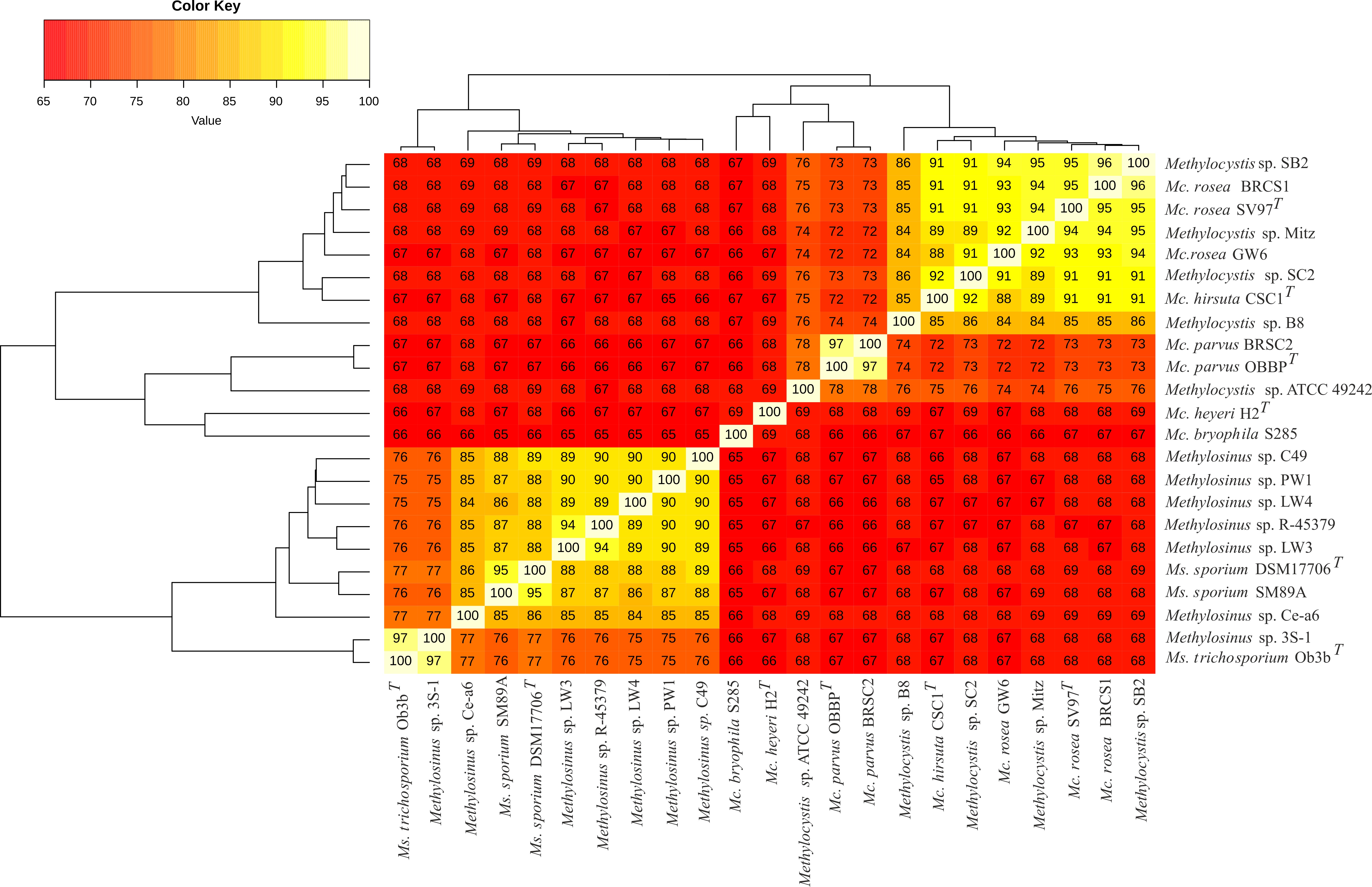

Supplement: Supplementary file 1 [file microorganisms-08-00768-s001.zip › Suppl_material/FigureS1.jpg]

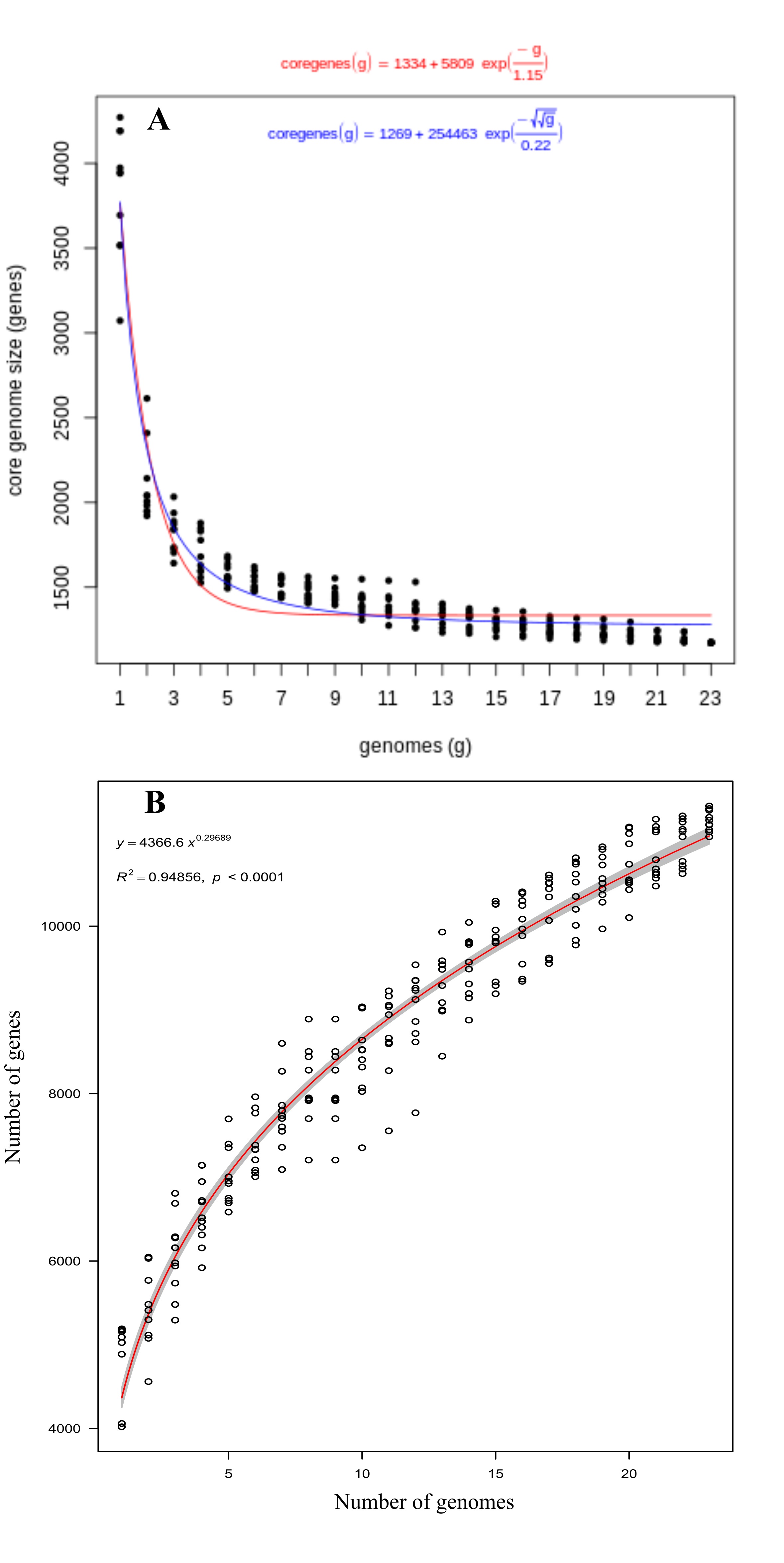

Supplement: Supplementary file 1 [file microorganisms-08-00768-s001.zip › Suppl_material/FigureS2.jpg]

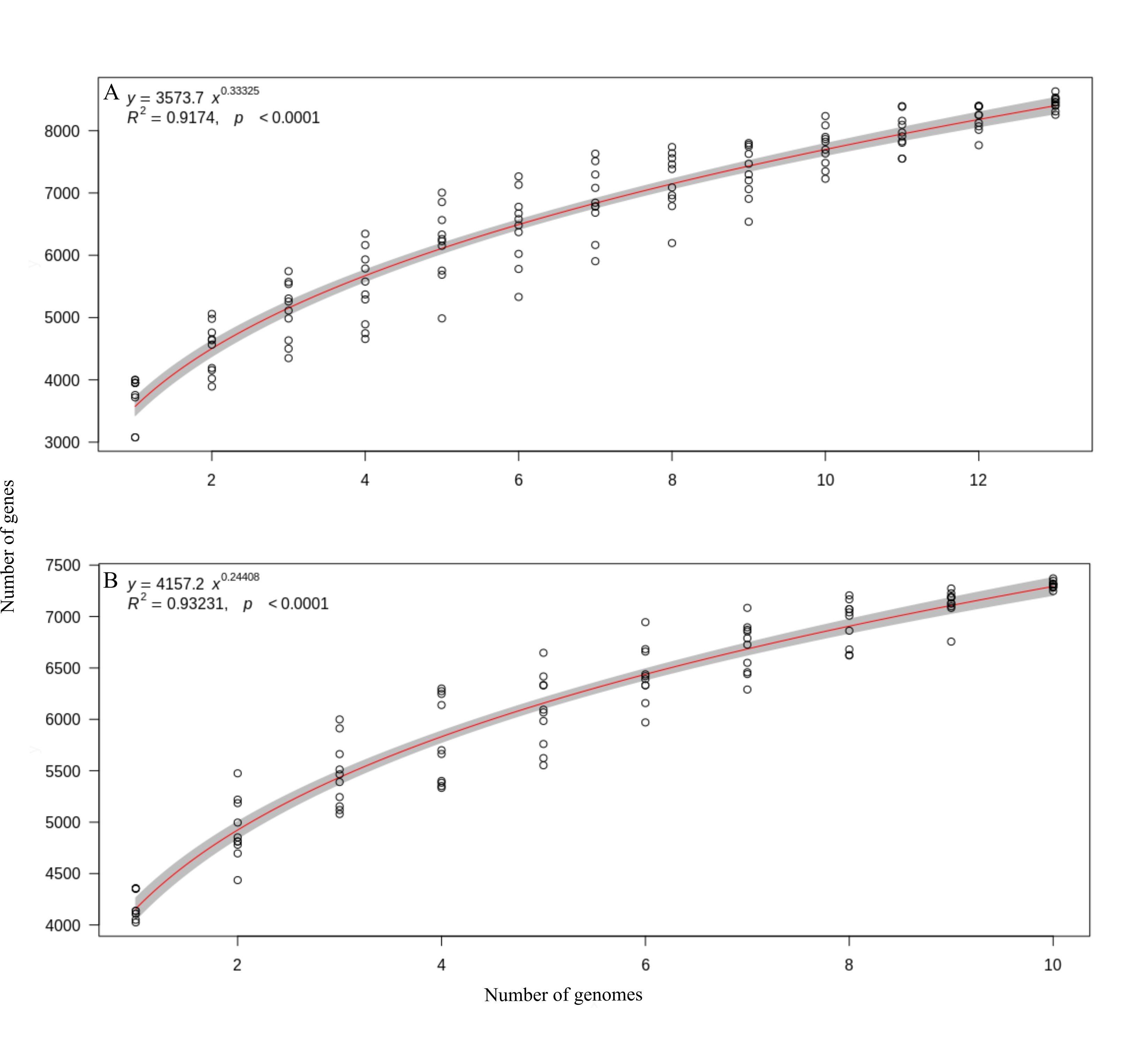

Supplement: Supplementary file 1 [file microorganisms-08-00768-s001.zip › Suppl_material/FigureS3.jpg]

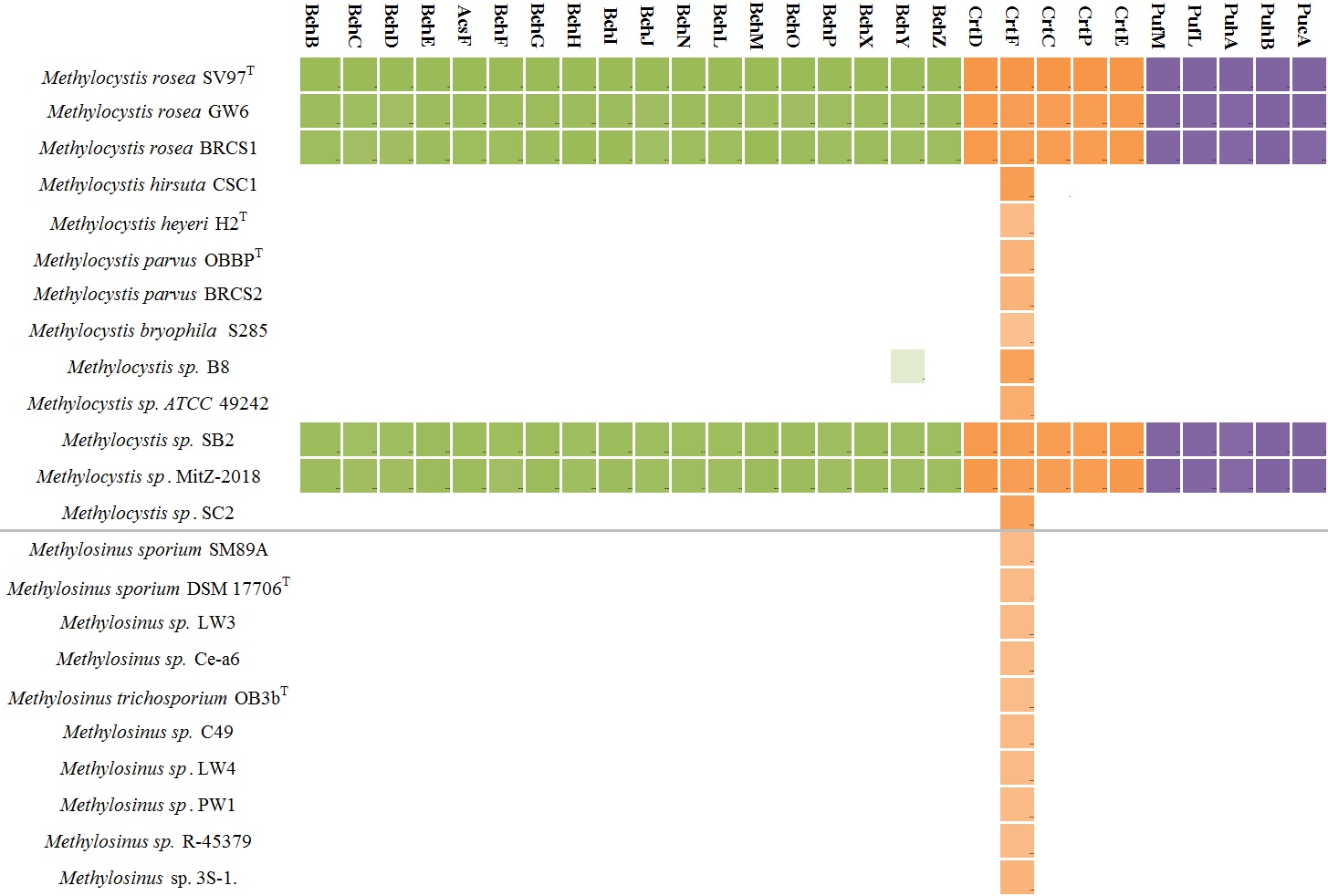

Supplement: Supplementary file 1 [file microorganisms-08-00768-s001.zip › Suppl_material/FigureS4.jpg]
